# The Influence of Age on Notch1 and VEGF‐A Responses During Exercise

**DOI:** 10.1155/jare/8041676

**Published:** 2026-09-29

**Authors:** Brooke R. Shepley, Lana H. Yacoub, Nicholas J. Lester, Rian A. Grassi, Jessica N. Kenney, Kevin J. Milne, Anthony R. Bain

**Affiliations:** ^1^ Department of Kinesiology, Faculty of Human Kinetics, University of Windsor, Windsor, Ontario, Canada, uwindsor.ca; ^2^ Schulich School of Medicine and Dentistry, Western University, London, Ontario, Canada, uwo.ca; ^3^ Department of Nursing, Faculty of Nursing, University of Windsor, Windsor, Ontario, Canada, uwindsor.ca; ^4^ Department of Chemistry and Biochemistry, Faculty of Science, University of Windsor, Windsor, Ontario, Canada, uwindsor.ca

## Abstract

Notch1 and vascular endothelial growth factor‐A (VEGF‐A) are polyfunctional signaling molecules that can be activated in response to elevated shear stress. In nonpathological conditions, they are integral to healthy endothelium; however, it is unknown whether their responsiveness during aerobic exercise, a robust shear stimulus, is impacted by age. Accordingly, in this exploratory cross‐sectional study, concentrations of VEGF‐A and Notch1 extracellular domain (NECD) were quantified in twenty‐nine (13 females) healthy adults (mean age: 32 years, range: 20–65), using venous blood samples taken at rest, immediately postexercise (cycle V̇O_2_ peak test), and following 15 min of recovery. NECD concentrations remained stable across exercise (condition effect: *p* = 0.186); however, overall NECD was elevated with age (age effect: *p* = 0.024). Moreover, the change in NECD following exercise varied by age (age × condition interaction: *p* = 0.028) and was significantly associated with carotid intima‐media thickness (cIMT) (*p* = 0.005, r = 0.475). Conversely, no significant main effects were found for VEGF‐A (all *p* > 0.05), and VEGF‐A was not associated with cIMT (*p* = 0.283, *ρ* = −0.114). These results indicate that the Notch1, but not VEGF‐A, response to acute exercise is altered across adult aging, with cIMT showing a secondary association that potentially reflects underlying vascular structural influences.

## 1. Introduction

Aging is a primary risk factor for the development of cardiovascular disease (CVD), largely due to progressive impairments of the endothelium [1, 2]. These impairments are characterized by reduced nitric oxide (NO) bioavailability, increased oxidative stress, and low‐grade inflammation, which promote a proatherogenic environment [1, 3]. Moreover, aging is associated with structural vascular changes, such as increased stiffness and arterial wall thickening, as assessed by carotid intima‐media thickness (cIMT) [4]. These age‐related changes alter the frictional force of blood flow acting on the endothelium (i.e., shear stress) [5]. Beneficial forward‐moving (antegrade) shear is reduced in aging vessels, whereas maladaptive oscillatory shear is increased, promoting endothelial dysfunction [5].

The Notch1 signaling pathway is crucial for maintaining endothelial health. Cultured endothelial Notch1 has been shown to detect and respond to antegrade shear stress and is necessary for endothelial cell elongation, alignment in the direction of blood flow, suppression of endothelial cell proliferation, and maintenance of intact cell–cell gap junctions [6]. Upon activation, Notch1 is cleaved to form Notch1 extracellular domain (NECD), which can be endocytosed into the ligand‐containing cell [7] or released into the circulation, where it can be measured [8]. The receptor is then sequentially cleaved, releasing the Notch1 intracellular domain (NICD) into the nucleus, where it modulates gene expression [9]. Importantly, NECD was recently shown to increase by ∼50% in response to elevated forearm shear stress in humans [8], demonstrating its viability as an in vivo marker of Notch1 activation.

Notch1 is a primary regulator of angiogenic signaling through interactions with vascular endothelial growth factor‐A (VEGF‐A) and its receptor, VEGFR2 [9]. Comparable to Notch1, in vitro endothelial cell models of VEGF‐A and VEGFR2 demonstrated mechanosensitivity to shear stress, as evidenced by elevated VEGF‐A and increased expression of VEGFR2 [10]. Interestingly, angiogenesis is impaired in aging vessels, which may reduce VEGF‐A concentrations in response to shear stimuli in older populations [1].

Chronic exercise has been shown to preserve vascular health and mitigate age‐related impairments, as evidenced by improved endothelial dilatory capacity in older athletes compared to age‐matched control subjects [11], possibly due to increased NO [12]. NO, which is reduced in aging vessels, interacts with the Notch1 pathway. Specifically, NO has been shown to regulate Notch1 signaling in human aortic interstitial and endothelial cells; therefore, reduced NO with age may affect Notch1 and its downstream targets [13]. As such, physical activity may improve vascular function, at least in part, through interactions between NO and Notch1 signaling. In addition to phenotypic endothelial modifications, exercise acutely alters systemic shear stress [14], which could stimulate Notch1 activity. However, aging vasculature alters shear patterns, potentially affecting Notch responsiveness in older adults compared with younger adults.

While augmented Notch1 activity has been associated with improved vascular health in mice [6] and has been shown to respond to acute increases in antegrade shear in humans [8], the effect of aging vasculature on this response remains unclear. Recent work from our lab demonstrated that circulating NECD was significantly associated with aerobic fitness (V̇O_2_ peak) but not with flow‐mediated dilation in young, healthy adults [15]. However, the extent to which this relationship persists with alternative forms of shear stress, such as maximal exercise, whether exercise elicits a distinct Notch1 response, whether that response varies across ages, and whether age‐related vascular remodeling, as assessed by cIMT, is associated with NECD and VEGF‐A responses remain to be determined.

Accordingly, the primary aims of this study were (1) to determine whether graded maximal exercise (cycle V̇O_2_ peak test) alters circulating concentrations of NECD and VEGF‐A and (2) to determine whether age and aerobic fitness are associated with changes in NECD and VEGF‐A across graded maximal exercise. A secondary, exploratory aim was to determine whether cIMT, which increases with age, is associated with changes in NECD and VEGF‐A following exercise.

## 2. Methods

An exploratory cross‐sectional study was conducted. Twenty‐nine (13 females) healthy adults (mean age: 32 years, range: 20–65) participated in this study. Participants were recruited through university postings, social media advertisements, and word‐of‐mouth. Participants were screened using a medical history questionnaire to confirm that they were free from overt CVD and medications that affected the cardiovascular system, were normotensive (118 ± 14/72 ± 11 mmHg), nonobese (24 ± 3 kg/m^2^), and had no history of smoking in the last 12 months. Females were required to be either premenopausal or 2 years postmenopausal. All female participants recorded the date of their last menstrual period; however, testing was not standardized according to menstrual cycle phase. Younger adults were required to be 18–30 years old, middle‐aged adults 31–49 years old, and older adults ≥ 50 years old. Participant characteristics, segmented by group, are shown in Table 1. The Research Ethics Board at the University of Windsor (REB no. 42638) approved all experimental procedures and protocols in adherence with the principles of the Tri‐Council Policy Statement and the Declaration of Helsinki, except for registration in a clinical database. Both written and verbal consent were obtained following a briefing of the experimental protocol (Figure 1).

**TABLE 1:** Participant characteristics.

	Young	Middle	Older	Total
Age (y)	23 ± 3	36 ± 7	56 ± 5	32 ± 14
*N*	17	6	6	29
Sex	9 M/8 F	3 M/3 F	4 M/2 F	16 M/13 F
HR (bpm)	68 ± 13	66 ± 11	59 ± 10	66 ± 12
SBP (mmHg)	118 ± 12	106 ± 14	127 ± 16	118 ± 14
DBP (mmHg)	70 ± 11	73 ± 14	78 ± 10	72 ± 11
BMI (kg/m^2^)	25 ± 4	24 ± 2	24 ± 3	24 ± 3
cIMT (mm)	0.5 ± 0.1	0.5 ± 0.1	0.8 ± 0.2	0.5 ± 0.2
V̇O_2_ peak (mL/kg/min)	40 ± 8	36 ± 8	33 ± 14	37 ± 9
Peak power output (W)	254 ± 61	231 ± 72	215 ± 114	241 ± 75
Exercise time (min)	11 ± 2	9 ± 1	11 ± 3	11 ± 2

*Note.* Data are presented as mean ± SD.

Abbreviations: BMI, body mass index; cIMT, carotid intima‐media thickness; DBP, diastolic blood pressure; HR, heart rate; SBP, systolic blood pressure.

**FIGURE 1:**
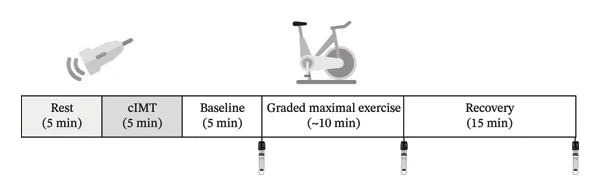
Experimental protocol and timeline. The vacutainer tubes denote when blood samples were taken.

### 2.1. Experimental Design

Participants arrived at the laboratory having abstained from alcohol, caffeine, and vigorous exercise for at least 12 h before testing, in addition to following an overnight water‐only fast. Participants were also not sleep deprived (average ≥ 7 h/night), as confirmed by the medical history questionnaire. Trials started between 8:00 a.m. and 10:00 a.m. On the day of testing, a venous catheter was inserted into a superficial antecubital vein. Blood samples were taken at baseline, immediately after the graded maximal exercise test, and 15 min following exercise (recovery). Immediately after exercise, participants completed approximately 1 min of active recovery, followed by seated recovery for the remainder of the 15‐min period.

#### 2.1.1. Peak Exercise Test

Incremental exercise to maximum was completed on a cycle ergometer (Ergoselect 100, Ergoline). Open‐circuit spirometry was used to measure respiratory gases (Quark CPET, COSMED, Chicago, IL, USA). Participants completed either a 20 W, 25 W, 30 W, or 35 W protocol based on their physical fitness as determined by a physical activity history questionnaire. There was a two‐minute warmup followed by increments of the determined wattage each minute until exhaustion while maintaining 60–80 rotations per minute (RPM). This was followed by a two‐minute cool‐down (RPM < 50). Maximal exertion was determined when participants failed to maintain ≥ 60 RPM and met at least one of the following criteria: 1) a respiratory exchange ratio of ≥ 1.15 and 2) a plateau in heart rate or V̇O_2_ despite an increased workload.

#### 2.1.2. cIMT

cIMT was measured as an index of structural vascular aging. Participants rested supine for 5 min with their necks in a neutral position. The common carotid artery was imaged in the longitudinal plane using B‐mode ultrasound (T3200, Terason, Burlington, MA, USA). Measurements were obtained from the far wall of the common carotid artery, approximately 1 cm proximal to the carotid bulb, on frozen images using the ultrasound’s built‐in calipers. The average of three measurements was recorded.

### 2.2. Quantification of NECD and VEGF‐A

Whole blood was collected in sodium citrate tubes (Vacutainer, cat. no. 363080, BD Biosciences, San Jose, CA, USA) and centrifuged at 1500 g for 10 min. Plasma was extracted and immediately stored at −80°C. The plasma samples were used to determine the concentration of NECD using commercially available immunosorbent assays (RayBio, cat. no. ELH‐NOTCH1, RayBiotech Life, Peachtree Corners, GA, USA), with an intra‐ and interassay coefficient of variation of < 10% and < 12%, respectively. The minimal detectable concentration was 20 pg/mL. VEGF‐A was quantified using commercially available immunosorbent assays (Bio‐Techne, cat. No. DVE00, USA R&D Systems Inc., Minneapolis, MN, USA), with an intra‐ and interassay coefficient of variation of < 7% and < 9%, respectively. The minimal detectable concentration is < 9.0 pg/mL. Samples were read according to kit instructions using a microplate reader (BioTek Synergy HT, BioTek Instruments, Winooski, VT, USA) and Gen5 software (Version 1.11, BioTek Instruments).

### 2.3. Statistical Analyses

Data are presented as means ± standard deviation (SD). Linear mixed models were used to examine circulating NECD and VEGF‐A across the exercise (baseline, immediately postexercise, and 15 min postexercise). Condition (time), V̇O_2_ peak, and age were included as fixed effects, along with the age × condition interaction; participant ID was included as a random intercept to account for repeated measures within individuals. Sex was also initially included as a fixed effect in the linear mixed models. However, it was not a significant predictor and did not influence the primary findings, so it was excluded to avoid unnecessary model complexity. Correlations were determined using Pearson’s correlations or Spearman’s when applicable. Due to the small and unequal sample sizes across age groups, age was analyzed as a continuous variable rather than categorically. Tests for normality were confirmed using Shapiro–Wilk tests. Statistical outliers, identified as values exceeding twice the SD from the mean, were reassayed via ELISAs to ensure they were not due to technical errors. Once findings were validated, outliers were Winsorized (NECD: *n* = 4; VEGF‐A: *n* = 1) to preserve sufficient statistical power [16–18]. All statistical analyses were performed using JASP (JASP Team, Amsterdam, Netherlands) and Prism (GraphPad, Boston, MA, USA).

## 3. Results

### 3.1. NECD Across Exercise

Results from the linear mixed model, as shown in Table 2, revealed that circulating NECD concentrations did not significantly change with exercise across all participants (condition effect: *F* (2, 51.42) = 1.74, *p* = 0.186). However, there was a main effect of age (*F* (1, 26.55) = 5.75, *p* = 0.024), with higher NECD concentrations observed with increasing age. Moreover, there was a significant condition × age interaction (*F* (2, 51.55) = 3.86, *p* = 0.028), indicating that the NECD response across the protocol differed by age. That is, increasing age was associated with a greater change in NECD from baseline to immediately postexercise (*p* = 0.008), whereas the change from baseline to recovery was not significantly altered by age (*p* = 0.390). A main effect of V̇O_2_ peak (*F* (1, 26.72) = 3.26, *p* = 0.082) failed to reach significance.

**TABLE 2:** Linear mixed model results for NECD and VEGF‐A across the protocol.

Biomarker	Effect	df	F	*p*
NECD	Condition	2, 51.42	1.74	0.186
	Age	1, 26.55	5.75	0.024^∗^
	V̇O_2_ Peak	1, 26.72	3.26	0.082
	Age × Condition	2, 51.55	3.86	0.028^∗^

VEGF‐A	Condition	2, 52.05	0.27	0.765
	Age	1, 25.66	0.08	0.785
	V̇O_2_ Peak	1, 25.80	2.39	0.134
	Age × Condition	2, 52.10	0.04	0.963

*Note.* Fixed effects included condition (baseline, immediately postexercise, and 15 min postexercise), age, V̇O2 Peak, and the age × condition interaction.

^∗^denotes significance at *p* < 0.05.

### 3.2. VEGF‐A Across Exercise

As shown in Table 2, a linear mixed model revealed that circulating VEGF‐A concentrations did not significantly change across exercise (condition effect: *F* (2, 52.05) = 0.27, *p* = 0.765). There was no main effect of age (*F* (1, 25.66) = 0.08, *p* = 0.785) or V̇O_2_ peak (*F* (1, 25.80) = 2.39, *p* = 0.134), and no significant condition × age interaction was observed (*F* (2, 52.10) = 0.04, *p* = 0.963).

### 3.3. Associations Between the Change in NECD and VEGF‐A With cIMT

Age was significantly correlated with cIMT (*p* = 0.020, *ρ* = 0.385), supporting the relationship between aging and vascular structure. To investigate associations between NECD and VEGF‐A activity with age‐related vascular adaptations, cIMT was compared to the percentage change in NECD and VEGF‐A from BL‐Ex and BL‐Rec, as shown in Figure 2. The change in NECD from BL‐Ex was moderately (*r* = 0.475) and significantly (*p* = 0.005) correlated with cIMT. To confirm these findings, the associations with cIMT and the absolute changes in NECD from BL‐Ex, as well as baseline NECD, were examined. The associations with cIMT remained significant for the absolute change in NECD (*p* = 0.015, *ρ* = 0.411) but not for baseline NECD (*p* = 0.132, *ρ* = 0.219). However, NECD from BL‐Rec was not correlated with cIMT (*p* = 0.074, *ρ* = 0.293). The changes in VEGF‐A from BL‐Ex and BL‐Rec were not significantly associated with cIMT (*p* = 0.283, *ρ* = −0.114; *p* = 0.418, r = 0.043, respectively).

**FIGURE 2:**
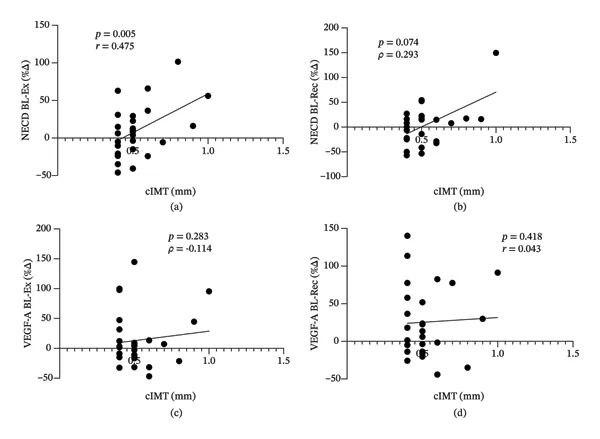
Relationships between carotid intima‐media thickness (cIMT; *x*‐axis) and the changes (%Δ) in NECD and VEGF‐A (*y*‐axis). (a) NECD from baseline to immediately post‐V̇O_2_ peak test (BL‐Ex; *n* = 28); (b) NECD from baseline to recovery (BL‐Rec; *n* = 26); (c) VEGF‐A from BL‐Ex (*n* = 28); and (d) VEGF‐A from BL‐Rec (*n* = 26). Two participants were unable to complete the recovery blood draw due to feeling unwell following the V̇O_2_ peak test.

## 4. Discussion

The overarching purpose of this study was to investigate the effects of age on concentrations of NECD and VEGF‐A across a single bout of graded maximal exercise. The primary findings of this study are that (1) circulating NECD and VEGF‐A concentrations did not significantly change across exercise; (2) NECD concentrations were influenced by age, but not aerobic fitness; and (3) cIMT was significantly associated with changes in NECD, but not VEGF‐A. These findings indicate that the Notch1 response to acute exercise, but not VEGF‐A, is altered across adult aging, with cIMT emerging as an important secondary correlate beyond aerobic fitness. This suggests that underlying vascular structural influences—namely, diffuse arterial wall thickening and/or subclinical atherosclerosis—may contribute to the age‐related differences in Notch1 responses.

### 4.1. Influence of Age and Fitness on NECD and VEGF‐A Activity

Age modified overall NECD concentrations in response to an acute bout of aerobic exercise but was unrelated to aerobic fitness. This is partially convergent from our previous study, where we demonstrated that changes in NECD across an FMD protocol were significantly associated with V̇O_2_ peak in young, healthy individuals [15]. However, this discrepancy is likely due to the different stimuli (i.e., FMD vs. exercise). Nevertheless, increasing age was associated with a greater change in NECD from BL‐Ex, whereas the changes in NECD from BL‐Rec did not vary with age. This indicates that the immediate NECD response from baseline to exercise became increasingly positive with age. While the exact mechanisms underlying the altered NECD responsiveness with age are unknown, one possibility is a difference in NECD shedding. Specifically, this may reflect elevated Notch1 receptor activation, leading to increased circulating NECD. Alternatively, this may be due to ligand‐independent shedding, leading to elevated NECD without necessarily activating the Notch1 signaling pathway [19]. However, receptor density and cleavage were not measured in the present study.

In contrast to NECD, VEGF‐A was not significantly altered in response to exercise or influenced by age or aerobic fitness. Previous research on VEGF‐A and VEGFR2 responsiveness to increased shear stress was observed in vitro [10]; therefore, in vivo signaling may require additional stimuli beyond acute shear to elicit a measurable change. In addition to shear stress, VEGF‐A is highly regulated by hypoxia [20]. As such, VEGF‐A may be more responsive to hypoxia than to shear stress in vivo, and exercise may not have induced a sufficient local hypoxic stimulus. This was demonstrated in Pramkratok et al. [21], whereby VEGF‐A increased following sprint training in hypoxic, but not normoxic conditions. Moreover, VEGF‐A has been shown to be more responsive to high‐intensity lower‐limb resistance training than to aerobic training in healthy adults [22]. Consequently, the VEGF‐A pathway may have required a stronger or separate stimulus to detect any measurable changes. Additionally, circulating VEGF‐A concentrations are known to be delayed, for example, in stroke, they tend to rise within 24 h and reach a peak at 7 days following the event [23]. Therefore, additional measures beyond 24 h after exercise might be necessary to detect delayed release.

### 4.2. Positive Association Between NECD and cIMT, but Not VEGF

Aging is associated with structural vascular changes, as reflected by increases in cIMT [4], which is supported by our data. In the present study, cIMT was approximately 0.3 mm larger in older compared with younger participants. Interestingly, greater cIMT was correlated with a larger NECD response from BL‐Ex. This relationship between cIMT and NECD activity suggests that vascular aging may influence the circulating NECD response pattern to an acute bout of aerobic exercise, potentially through vascular remodeling. Although the influence of Notch1 signaling appears to be context dependent [6, 24, 25], in, otherwise, “healthy” models, this pathway promotes endothelial quiescence and is atheroprotective [6, 26]. Therefore, the altered circulating NECD response to aerobic exercise with age, and its positive correlation with cIMT, may reflect a compensatory response to elevated shear stress. Specifically, aging vasculature may increase Notch1 activity through enhanced receptor‐ligand interactions (e.g., increased receptor expression, ligand presence, or both) as an adaptive response to age‐related vascular remodeling (e.g., increased stiffness). However, this remains speculative as shear stress, receptor expression, NICD, and receptor–ligand interactions were not assessed in the present study.

In contrast to NECD, VEGF‐A activity was not associated with cIMT, likely due to the negligible change in VEGF‐A across the protocol. However, lower plasma concentrations of VEGF‐A were associated with reduced cIMT in metabolically unhealthy obese individuals, but not metabolically healthy obese individuals [27]. Accordingly, VEGF‐A may be more responsive in proinflammatory or metabolically dysregulated environments. This is consistent with our findings, as the participants in this study were metabolically healthy, albeit nonobese, which may partially explain why VEGF‐A activity remained stable.

### 4.3. Considerations

Several factors should be considered when interpreting the external validity of the present findings. First, the recruitment strategy was largely from the Department of Kinesiology at the University of Windsor, resulting in a potentially more “health‐conscious” group compared to the broader population. Participants were generally healthy, nonobese, normotensive adults without overt CVD, and, therefore, these findings may not be generalizable to individuals with cardiovascular or cardiometabolic conditions. Moreover, the number of participants within each age group was uneven; therefore, distinct group comparisons could not be conducted. Larger studies with more balanced age groups and greater representation of adults aged ≥ 65 years should be performed to confirm the present findings. Although the graded maximal exercise test intensity was individualized to fitness levels, the overall workload was generally lower with age, which may have contributed to age‐related findings. We did not account for the menstrual phase in female participants or exclude contraceptive use. Although no sex differences were observed, testing was not standardized to menstrual cycle phase; therefore, hormonal fluctuations may have contributed to the responses. Premenopausal and postmenopausal women may differ in their responses to acute exercise; however, with only two postmenopausal participants, comparing menopausal status was not feasible. Similarly, due to the limited sample size of older participants, the independent effects of age and menopausal status could not be distinguished. Additionally, NECD and VEGF‐A were assessed in the venous circulation, whereas cIMT is an arterial measure; thus, the results may not directly reflect mechanosignaling pathways in the arterial vascular bed. Plasma volume was not measured in the present study. Although the exercise duration was relatively short, exercise‐induced hemoconcentration may have influenced circulating concentrations of NECD and VEGF‐A. Future research should consider measuring shifts in plasma volume to account for the potential influence on circulating biomarker concentrations. Shear stress was also not measured in the present study; therefore, the responses of Notch1 and VEGF‐A cannot be directly attributed to changes in shear stress. It is important to recognize that circulating NECD is an indirect marker of Notch1 activation and should, therefore, be interpreted with appropriate caution. Nevertheless, it currently represents the only practical, minimally invasive approach for assessing systemic Notch1 mechanosignaling in vivo. Lastly, these findings should be interpreted within the context of a relatively short (∼12 min) bout of maximal exercise. Longer‐duration exercise, and the accompanying greater cumulative shear stimulus [6, 8], may elicit different Notch1 and VEGF‐A responses.

## 5. Conclusion

In conclusion, NECD and VEGF‐A were not significantly increased in response to acute (∼12 min) aerobic exercise. However, under the present exploratory experimental conditions, the NECD response pattern, but not VEGF‐A, was altered with age, and the change in NECD measured at rest and immediately at the termination of exercise was positively associated with cIMT. These findings suggest that age‐related differences in mechanosignaling responses to acute exercise may be related to altered vascular structure.

## Author Contributions

Anthony R. Bain conceived the study. Brooke R. Shepley, Kevin J. Milne, and Anthony R. Bain analyzed the data. Brooke R. Shepley and Anthony R. Bain drafted the study. Brooke R. Shepley, Lana H. Yacoub, Nicholas J. Lester, Rian A. Grassi, Jessica N. Kenney, and Anthony R. Bain contributed to data collection. All authors revised and contributed to the final version of the manuscript.

## Funding

This work was supported by the Natural Sciences and Engineering Research Council of Canada (NSERC) Discovery Grant, held by Dr. Bain (RGPIN‐2020‐05760).

## Conflicts of Interest

The authors declare no conflicts of interest.

## Data Availability

The data that support the findings of this study are available on request from the corresponding authors. The data are not publicly available due to privacy or ethical restrictions.
